# Adaptation under strain: an ethnographic process evaluation of community-based psychosocial support services for refugee claimants during the height of the COVID-19 pandemic in Montreal, Canada

**DOI:** 10.3389/fpubh.2023.1143449

**Published:** 2023-05-30

**Authors:** Yufei Mandy Wu, Aseel Fawaz Alzaghoul, Leonora Indira King, Rosy Kuftedjian, Rachel Kronick

**Affiliations:** ^1^Division of Social and Transcultural Psychiatry, Department of Psychiatry, Faculty of Medicine and Health Sciences, McGill University, Montreal, QC, Canada; ^2^Centre for Child Development and Mental Health, Lady Davis Institute, Montreal, ON, Canada; ^3^Lady Davis Institute, Jewish General Hospital, Montreal, QC, Canada; ^4^Institut Universitaire SHERPA, Montreal, QC, Canada

**Keywords:** COVID-19 pandemic, asylum seekers, community health services, psychosocial support, internet-based intervention

## Abstract

During the first 2 years of the COVID-19 pandemic in Canada, tens of thousands of refugee claimants faced worsened resettlement stress with limited access to services. Community-based programs that address social determinants of health faced significant disruptions and barriers to providing care as a result of public health restrictions. Little is known about how and if these programs managed to function under these circumstances. This qualitative study aims to understand how community-based organizations based in Montreal, Canada, responded to public health directives and the challenges and opportunities that arose as they attempted to deliver services to asylum seekers during the COVID-19 pandemic. We used an ethnographic ecosocial framework generating data through in-depth semi-structured interviews with nine service providers from seven different community organizations and 13 refugee claimants who were purposively sampled, as well as participant observation during program activities. Results show that organizations struggled to serve families due to public health regulations that limited in-person services and elicited anxiety about putting families at risk. First, we found a central trend in service delivery that was the transition from in-person services to online, which presented specific challenges including (a) technological and material barriers, (b) threats to claimants’ sense of privacy and security, (c) meeting linguistic diversity needs, and (d) disengagement from online activities. At the same time, opportunities of online service delivery were identified. Second, we learned that organizations adapted to public health regulations by pivoting and expanding their services as well as fostering and navigating new partnerships and collaborations. These innovations not only demonstrated the resilience of community-organizations, but also revealed tensions and areas of vulnerability. This study contributes to a better understanding of the limits of online service delivery for this population and also captures the agility and limits of community-based programs in the COVID-19 context. Its results can inform decision-makers, community groups and care providers to develop improved policies and program models that preserve what are clearly essential services for refugee claimants.

## 1. Introduction

The COVID-19 pandemic has had profound health and psychosocial impacts globally and made stark some of the sociopolitical inequities that drive health disparities. Asylum seekers are an example of a population who had been disproportionately affected, as they faced mounting barriers in accessing healthcare, economic resources, education and social support, all of which had a cumulative effect on their physical and mental health (1, 2). Asylum seekers, also known as refugee claimants, are people who arrive in a host country seeking protection from violence, war or persecution in their country of origin. Unlike refugees who arrive with a secured path to citizenship, or, more recently, Ukrainian refugees who are offered temporary protection in most countries, refugee claimants must undergo an often-protracted process to determine if they meet criteria for refugee status. In Canada, for example, the process takes an average of 2 years (3). The vast majority of asylum seekers are racialized, and from low and middle-income countries (4). Even when arriving in a high-income country seeking protection the limbo and uncertainty claimants live with was significantly worsened during the pandemic, as they faced closed borders and limited access to services that were deemed inessential following public health regulations and lockdowns, further entrenching their social exclusion (5).

Refugees and asylum seekers struggle with various post-migration difficulties that have been identified as salient predictors of mental health and well-being (6–9). These stressors include socioeconomic factors such as poverty, unemployment (10), insecure housing (11), and language barriers (12); as well as social factors such as loneliness, social exclusion (8, 13), racism and discrimination (14, 15). Research has now shown that for asylum seekers these post-migration difficulties were significantly exacerbated by the pandemic, compromising their mental health and increasing risk of suicide (16, 17). Asylum seekers’ living and working conditions – as security guards, custodians in hospitals, and personal care attendants in long-term care facilities – deepened their isolation due to shift work and fears of contamination and put them at higher risk of contracting COVID-19 (18, 19). Unlike many host-country peers who readily transitioned to online spaces to seek social support, claimants had no straight-forward solution to combat isolation because of lack of access to technology and closure of public spaces (2). Meanwhile, many asylum-seekers frontline workers who were risking their lives were also facing significant uncertainty and worries over imminent deportation which increased depression, anxiety, insomnia, and psychological distress (20).

While stressors and ensuing mental health problems faced by asylum seekers were clearly augmented by the pandemic (21), refugee claimants continued to face barriers to accessing support and healthcare (22, 23). There is well-elucidated literature on the difficulties asylum claimants face in accessing health care, related to challenges with trusting and navigating the system, discrimination from healthcare providers and institutions, and cultural and linguistic barriers (16, 24–31). The pandemic further deprived claimants of healthcare access and increased barriers to information and service access. For example, one Canadian study found that immigrants and refugees in particular faced disruptions in accessing basic resources (such as personal protective equipment and food delivery) and health and social support services (such as banking and immigration resources) which hindered their resettlement (32). Emerging data also suggest that refugees and asylum seekers avoided seeking care due to fear of infection and lack of information on available services (17, 33–35). These limitations were at times attributed to limited internet and English proficiency (32, 36). The United Nations Network on Migration (37) deemed refugee and migrant communities to be the “hardest to reach” in the dissemination of information regarding COVID-19.

For refugee claimants, social services are crucial to accessing information and building trust in healthcare professionals and healthcare systems. Community-based organizations, specifically, their psychosocial components play a vital role in refugee claimants’ resettlement, often acting as a bridge between vulnerable populations and institutions by providing accessible informational, instrumental, and social support by drawing on an ecological perspective that transcends traditional mental health services which focuses on individualistic health care provision (38, 39). In both low and -middle income countries (LMIC) and higher income countries (HIC), the work of community-based organizations proved to be especially vital during the COVID-19 pandemic. One team of healthcare providers in London, UK collaborated with community organizations to build an integrated model of care that centralized health and social services to address the complex needs of asylum-seeking families (40). In Malaysia, community-based organizations distributed essential food packages across networks connected by local community leaders (41). The UNHCR in several countries collaborated with community volunteers as part of their community-based protection work, which ensured information on service access was available within the community. For example, in Ethiopia, South Sudan and Bangladesh, refugee representatives, secondary school students and community health workers played an essential role in distributing information and essential hygiene items, demonstrating basic hygiene practices such as handwashing, and organizing food distribution in smaller groups (42). However, the essential role community services fulfill was often unrecognized, as some of the community-based initiatives were forced to halt, limit, or reinvent their services in response to public health measures (43). How and if programs managed to continue supporting refugee claimants is still under-explored. Yet, understanding is critical, on the one hand, to paint a clearer picture of the potential collateral effect of public health measures on refugee claimants, and on the other, to better prepare practitioners and public health decision-makers in case of future public health emergencies.

This qualitative study thus aims to understand how community-based organizations in Montreal, Canada responded to public health directives and the challenges and opportunities that arose as they attempted to deliver services to asylum seekers during 1 year of the COVID-19 pandemic. By illustrating both service providers’ experiences with service delivery and refugee claimants’ experiences in using these services during the pandemic, we aim to provide a full picture of how the pandemic affected these organizations that are providing essential services to refugee claimants. More specifically, we explored how service providers coped and adapted to the challenges imposed by the pandemic; what supported or hindered their work, and how the impact to their services in turn affected refugee claimants’ resettlement.

## 2. Methodology

### 2.1. Methodological approach

We used an ethnographic framework which is highly compatible with a process evaluation approach (44) and allowed us to observe how programs and practitioners experienced the changes in their organizations, their work and the provision of services. In asking what is happening in the adaptation and closure of services, we were able to generate contextualized data reflecting the meaning and consequences of interventions (or lack thereof) that captured the perspectives of those who are both delivering and receiving the services. As *critical* ethnography (45), we were attentive to issues of power, inequitable distribution of resources, and embodied exclusion, and sought to understand how structures, institutions and discourses reproduced or resisted oppressive frameworks and practices.

In addition, we draw on notions from ecosocial theory to orient our understanding of individual and collective experiences of asylum-seeking families and service providers working with them during the pandemic (46). Ecosocial theory was developed by Nancy Krieger in 1994 (47) to conceptualize ‘health inequities in relation to power, levels, life course, historical generation, biology, and ecosystems’ (48). An ecological framework has also been mobilized to conceptualize a holistic model of recovery for asylum seekers after experiences of war and collective trauma (49). While we focus less on embodiment and biology, an ecosocial framework allows us to explore how asylum seekers’ wellbeing is impacted by the ecological context of the COVID-19 pandemic at individual, communal, and societal levels (46, 50). Further, an ecological framework helps us take into account the structural, interpersonal and individual interrelated factors shaping community service providers experiences and organizational practices during the pandemic. By understanding both service delivery and lived experiences through an ecosocial frame, we can better capture the interplay of factors at work for community organizations that protect asylum seekers’ health, with the aim of informing preventive approaches for future public health crisis (51).

### 2.2. Methods

#### 2.2.1. Study setting and participants

This study is nested within a larger interdisciplinary investigation of the implementation, adaptation, and impacts of a two-year community-based, multi-site, psychosocial support program for asylum-seeking families in Montreal called “Welcome Haven.” The Welcome Haven collaborates with several community- and public-sector organizations to provide accessible and comprehensive support to asylum-seeking families. The Welcome Haven was launched in September 2021, just prior to provincial directives for re-closure during the initial Omicron wave.

There were two groups of participants in the current study. First, service providers over 18 years of age from organizations who had first-hand experiences in delivering services to asylum seekers during the pandemic and who had actively collaborated with the Welcome Haven program between September 2021 and April 2022 just prior to and during the Omicron wave in Canada were selected through purposive sampling. Second, we recruited asylum seeking mothers over 18 years of age who had participated in at least two workshops at Welcome Haven, whether online or in-person. Only service providers who spoke English or French fluently were recruited, whereas for claimants there was no language restriction and interpreters were used when needed. For both groups of participants, we used a purposive sampling strategy (52), rather than a statistical one, aiming to capture a range of experiences across key service organizations and refugee claimant mothers. In total, nine service providers across seven different community organizations (including Welcome Haven) in Montreal and 13 refugee claimants were included in our study.

#### 2.2.2. Data collection

Data were primarily generated through in-depth semi-structured interviews with participants, ranging from one to two and a half hours. These interviews were conducted via Zoom and were audio recorded and transcribed verbatim. All records and transcripts were stored in a secure drive that could only be accessed by the research team. Ethical approval to conduct these interviews was obtained from the Research Ethics Committee of the CIUSSS du Centre-Ouest-de-l’Île-de-Montréal, project reference number 2021–2461. Written consent was obtained from all participants, who were given the time to read the form and ask questions. A compensation of $15 in the form of gift cards was given to each participant.

The interviews with service providers tried to elicit the challenges and opportunities in responding to asylum-seeking families’ needs before and during the COVID-19 pandemic as well as reflections on the programs offered, organizational adaptations and expectations for post-pandemic service delivery. Interviews with asylum claimants aimed to generate understanding of their lived experiences of the pandemic in Montreal between September 2021 and April 2022, and to understand how services were available, accessed, and meaningful to them.

The second means of data collection was participant observation. Researcher-practitioners in the Welcome Haven who worked collaboratively with multiple community-based services to deliver psychosocial support workshops during the pandemic generated extensive field notes over a period of 1 year (2021–2022). The field notes were collected by multiple actors in the Welcome Haven project, including creative expression therapists who facilitated workshops, graduate students who conducted interviews and also supported in-person and online workshops, and the principal investigator. These fieldnotes documented the process of implementation, adaptation and delivery of services as well as the process of collaboration across organizations and sectors. The notes also reflected on individuals’ experiences of delivering care in the context of the second year of the COVID pandemic.

#### 2.2.3. Data analysis

Data were then analyzed inductively and deductively using thematic content analysis (53). Two research assistants conducted a preliminary analysis of interviews and field notes independently to identify the main themes, which were then discussed and revised for an initial coding map. All the interviews were subsequently coded using NVivo according to the map, with refinements in coded themes elaborated in group discussions with the first and last author; Attention was given to contextualizing service providers’ perspectives with those of participants’ thus reflecting multiple perspectives.

## 3. Results

The claimant participants were all mothers, 9 out of 13 of whom were married with an average number of two children, and their average length of resettlement was around 8 months. Participating organizations’ psychosocial support services ranged from provision of orientation sessions, food and furniture donations, housing support, and individual mental health support. Table 1 details the main characteristics of the participating organizations and Table 2 outlines the demographic information of the claimant participants.

**Table 1:** Characteristics of the participating organizations.

Organization code	Role(s) of interviewee	Services offered	Duration of operation	Funding source
0428	Program Manager	Orientation session for newly arrived asylum seekers	3 years	Philanthropic funding
0511	Executive Director and Mental Health Coordinator	Range of services for both refugees and asylum seekers including academic aid, legal aid, advocacy center, language center, wellness and mental health sector, employability sector	6 years	University, federal, provincial fundings; private donations; endowment funds from international organizations
0513	Director for Emergency Services	Services provided to asylum seekers include donation of food and clothing	68 years	City and provincial funding; philanthropic funding; private donations
0518 and 0608	Director of Social Initiatives and Community Worker	Donation of furniture, social initiative including resource navigation	5 years	Private donations
0607	Program manager	Housing services for asylum seekers	5 years	Private donations
0712	Project manager	Services for asylum seekers include psychosocial and daycare services	17 years	City, philanthropic funding
0719 (Welcome Haven)	Research coordinator	Psychosocial support programs including informational and creative-expression workshops	1 year	Research, university funding

**Table 2:** Demographic information of the claimant participants.

Participant code	Region of origin	Length of resettlement (month)	Marital status	Number of children	Education level	Language
0324–01	Central Africa	7	Married	2	Professional	French
0324–02	Caribbean	16	Married*	1	Professional	French
0325	East Africa	36	Single	2	High school	English
0328	East Africa	4	Single	1	Professional	English
0402	West Africa	27	Single	2	Professional	English
0428	West Africa	2	Single	2	High school	English
0503	Central Africa	5	Married*	2	Professional	French
0504	South America	5	Married	3	Professional	Spanish
0516	West Africa	3	Married	2	Professional	English
0530	South America	1.5	Married	2	Professional	Spanish
0614	Central Africa	2	Married	3	High school	French
0617	Central America	4	Married	3	Professional	Spanish
0719	South America	3	Married	2	Professional	Spanish

* indicates married but the partner is still in the country of origin.

Results showed that many organizations struggled to serve families due to public health regulations that limited in-person services. A central trend in service delivery was the transition from in-person services to online, which presented specific challenges including: (1) difficulty in accessing claimants due to technological and material barriers, (2) threats to claimants’ sense of privacy and security, (3) difficulty in catering to a linguistically diverse population, and (4) disengagement from online activities. At the same time, opportunities of online service delivery, specifically in stress-relief and forging social connections, were identified. Beyond the transition to online services, organizations adapted to public health regulations by pivoting and expanding their services and fostering and navigating new partnerships and collaborations. Despite the barriers and strains imposed by the pandemic, organizations had demonstrated innovation and resilience during the adaptation process.

### 3.1. Experiences of delivering online services during the COVID-19 pandemic

#### 3.1.1. Technological and other material barriers

All of the organizations that were interviewed reported transitioning to online services (either completely or partially) to connect with the asylum-seeking community during the first 2 years of the pandemic. Service providers reported several technological challenges experienced by shifting informational or creative expression workshops online: distracting background noises, unstable internet connections, participants’ confusion over platform functions such as muting and unmuting microphones, turning videos on and off, and “hand-raising.” For asylum seekers, the most apparent barrier was limited financial resources that resulted in limited access to technological devices, such as computers, tablets, and access to cellular data. There seemed to be few remedies for this barrier. For example, the shelters where many asylum seekers resided when they first arrived in Montreal only had one designated area for Wi-Fi access, which was slow and often unstable. Libraries and other community spaces that normally offered access to the internet were also closed to the public during the pandemic; even when they reopened, their capacity remained very limited due to social distancing regulations, and asylum seekers were sometimes turned away from the libraries because of their immigration status (even though asylum seekers have the right to access library services). Service providers lamented these barriers in reaching asylum seekers who were most in need of information particularly during the pandemic:

[it] is frustrating, because we can't reach as many people when we're producing online materials. People don't have access to zoom if they don't have access to internet, if they don't have access to Wi-Fi, if they don't have a cell phone.

Some service providers responded to their clients’ lack of access to technology by acquiring donated devices: “I had to secure technological devices for everyone to be able to use in a very short period of time…we had to act really quick [to] give out as many laptops as we could.” Although the pressure of quickly having to secure laptops was challenging, service providers deemed this effort essential in facilitating service uptake for claimants and in expanding service reach for organizations.

Beyond technological access, online psychosocial workshop delivery required specific resources that were not readily available to asylum seekers. One organization gave the example of their attempt to host online art hives and cooking workshops despite participants lacking specific materials. The service provider responded to this issue by “having these [art and cooking] kits ready and sending them [by mail] beforehand.” On an organizational level, some providers reported an administrative overhaul to adapt to online service delivery, which was often time- and labor-intensive. For instance, one organization reported the demanding process of transferring clients’ files online and working from different portals to ensure confidentiality. All of these examples reflected the demand on financial and human resources in transitioning to online services so that service providers could continue to provide services to claimants in the context of pandemic regulations.

The technological and material barriers often appeared to impede social connection and even limit participants’ sense of emotional safety, especially during group activities with younger children (ages 5–11), causing frustrations for both participants and facilitators. At Welcome Haven, many children were unable to follow the online social communication cues that in-person presence made possible. One facilitator wrote in their fieldnotes about a young participant who was joyful and eager to share, “but it was hard to work as a group as she would just talk into the camera whenever she wanted, sometimes interrupting other kids, which seemed to have flustered her a little bit.” When facilitators used their host privilege to mute a participant after asking for their permission, the child would not know how to unmute themselves when they needed to speak. Given refugee claimant children are often victims of social violence and persecution in their countries of origin, issues of voice, silencing and witnessing were high stakes for the children’s sense of emotional safety in the workshops, which were proved difficult to protect online. In addition, children were also often distracted by other stimuli in their environment such as other family members in the background. Finally, unlike in our in-person workshops where children and parents could attend simultaneously but participate separately (in different rooms), we found facilitating group work with children online required constant parental supervision as parental assistance with using platform functions was necessary to ensure children’s participation.

#### 3.1.2. Privacy and sense of security

Another challenge in shifting services online was the assurance of privacy and security. In providing individual support, service providers described the delicate balance between creating confidential spaces and hosting them online: “Yes, online can give you that sense of ease of accessibility, but sometimes being in a house with a big family does not make it really easy to have a safe space to talk.” One service provider shared that this issue was especially salient for victims of domestic or intimate partner violence, who had to wait for a perpetrator to leave before they could call someone. Even if privacy was temporarily available the fear of a family member perpetrator returning at any moment interfered with their ability to reach out. In this sense, online service delivery posed some significant dangers from which refugee claimants could have been protected in an in-person encounter with a provider. Given rates of intimate partner violence increased in Canada and globally during the COVID-19 pandemic, this finding is in keeping with practitioners’ grappling with the protection of victims in online service delivery (54). Despite these potential dangers, other service providers also suggested that having the ability to connect to someone remotely, especially in emergencies, could be reassuring and even protective for claimants.

#### 3.1.3. Language barriers

The linguistic diversity of the asylum-seeking population demanded multilingual workshops that were hard to navigate online. One service provider observed that during in-person workshops, participants who shared the same language could sit together so that each facilitator could be designated to a different language group and interpret simultaneously. This was however not possible online, and instead some organizations had to divide informational workshops into three separate ones according to language, which in turn limited cross-cultural connections between asylum seekers, and increased the human resources required to deliver information.

#### 3.1.4. Disengagement in online activities

Despite the exacerbation of pre-existing social isolation experienced by asylum-seeking families during the pandemic, organizations observed participants’ reluctance to attend and engage in online activities. For Welcome Haven, even when a workshop was extensively shared and promoted online, at times reaching more than a thousand asylum seekers, only 8–10 participants would attend. Even when they did attend, most participants kept their cameras off. Fieldnotes suggest that only participants who had previously established rapport with service providers during in-person workshops turned on their cameras and engaged in small talk. Previous in-person participation in fact seemed to be the primary reason most people attended workshops online. One asylum-seeking mother shared in her interview: “I know your activities, your meetings are interesting, are informative. It will help [my child]. So without thinking, I accepted the invitation.” For another mother, however, while she was a frequent participant in our in-person workshops, she explained in the interview that she had never participated in the online workshop because she believed, “it’s very important not to be in front of a computer [when] your child is next to you.” This example suggests that lack of attendance in online workshops was intentional and not just a consequence of technological difficulties, especially for parents who already felt children were over-exposed to screens.

In coping with poor online attendance, we found reaching out to individual participants by phone through our collaborating organization to be the most effective recruiting method for online activities during the pandemic. Because we had operated in-person workshops for 2 months prior, we had existing connections with claimants whom we contacted personally to invite to the workshop, sometimes even 5 min before the workshop began. The research coordinator at Welcome Haven explained that calling individual participants allowed us to bridge the gap of social connection by casually checking in on the families and see how they were coping, thus signaling the sentiment of care which was meaningful to claimants and promoted a sense of integration. Although calling participants individually was time-consuming and laborious, our workshop facilitators noted in their fieldnotes that participants had expressed gratitude in these reminders because “the workshop link could have gotten lost” or they would “lose track of time.”

The struggle to reach and retain asylum seeker participants online was not unique to Welcome Haven. Another service provider who hosted psychosocial workshops online also shared that: “There are people who participate much less virtually in the sense that they are there, but they are not quite 100% there, camera closed, they do not participate.” Likewise, one creative expression workshop facilitator for Welcome Haven reflected in their fieldnotes on whether and how they could convince children participants to turn on their camera:

What do you do as a therapist [or facilitator] when someone refuses to turn on their camera? Do you let them be safe and invisible? Or do you challenge them to turn on their camera because other participants have it on and are being vulnerable?

Acutely aware of the limits to emotional safety that the online workshop space entailed, the facilitators grappled with how to respond to children’s–perhaps adaptive–desire to stay somewhat hidden which stood, it seemed, in contrast with the aspirations of the workshop to forge bonds that equalize power differences between children.

The lack of social connection through virtual platforms appeared to affect the program facilitators, who felt uneasy when they were the only ones to have their cameras on and questioned whether this dynamic created a power-imbalance online. This participant-observer noted in her fieldnote:

At in-person workshops we are divided into different spaces, so it’s not an overwhelming group of staff, and [thus] easier for us to fade into the background during presentation… whereas online the staff’s cameras are the only ones on, which makes our presence more obvious. But then when I turned off the camera, I felt weird about not contributing to the collective virtual space and it feels intrusive in a different way such that I’m a “faceless ghost” lurking.

While facilitators held power and privilege whether online or in-person, the online format made participant-observers feel that differences between asylum claimants’ and facilitators’ positions were starker and more problematic. As described above, facilitators felt they might contribute to a feeling of surveillance in the online space, and that staff stood out as Other than the asylum seekers. During in-person workshops, the Welcome Haven aims to create a participatory, collaborative space of mutual aid whereby asylum seekers themselves often facilitate, and staff sit with claimant families and interact together throughout, creating moments of witnessing and shared experience. The online workshops, on the other hand, seemed to create a unidirectional delivery of information and services and lacked moments of mutual connection. The absence of sustained rapport was particularly concerning when participants appeared visibly upset and distressed by program activities. Unlike in person workshops where the facilitator could respond by taking this participant away from the group for a discussion or break, this was not as easily facilitated online through “breakout rooms.”

Many service providers expressed a similar disinterest in online activities, sharing candidly their empathy toward families who disengaged online. One service provider expressed: “I would not have done any online activity if it was not for work.” Both participants and facilitators found online psychosocial programming to be much less engaging and motivating.

#### 3.1.5. Opportunities of online delivery

Despite the significant barriers and potential risks of online service delivery, interviewees also reported that effectively engage asylum seekers and build rapport was possible. One way workshop facilitators engaged participants was through incorporating certain online functions in activities, such as turning on and off audio or video to express agreement to statements. Fieldnotes suggested that once trust was established child participants were comfortable to playfully engage with the online features. For example, discussing the emotional impact of COVID, one participant used “angry, sick, and sad” emojis in the chat on the platform to express some of her feelings about the pandemic.

Successfully engaging participants during online workshops relied on service providers’ skills and creativity in developing activities that appealed to participants. For example, facilitators found that encouraging participants to use objects in their homes increased candid expressions of emotion. Fieldnotes described a woman lifting her chair and explaining that the chair was “tired of being in the same place!” Reflecting on the experience, this mother shared in her interview: “[Attending this workshop] makes me feel relieved because seeing people sharing jokes, sharing experiences, […] it helps [with] stress, loneliness [from] the work I do at home […] It makes me have this sense of relief.” Another way for participants to alleviate stress in online workshops was through the use of music. Facilitators described participants’ responses to music: “we are all grooving, finding ways to make it connect, play on camera, with the kids, with lamps.” One participant shared in her interview that she would not have danced or sang if the workshop was happening in-person, and it was only because she was at home that she was able to open up.

Beyond stress-relief, online workshops also had the potential of forging social connections that claimants described as existentially meaningful. At one of the online psychosocial workshops, only one participant attended. Nonetheless, this participant shared in her interview that after attending this online activity and receiving individual support:

We were very happy, because we felt finally, we would have somebody to talk to. Somebody was going to help us, somebody was going to give us a hand, somebody would listen to us. At last, we were—we existed—we were going to exist for someone.

This participant arrived in December 2021, at the height of the Omicron wave in Quebec, hence she had never had access to any in-person services. She expressed feelings of intense loneliness and helplessness at the start of her resettlement, making the assistance extended by community programs, albeit online, meaningful. Notably, organizations were only able to offer these online opportunities because of facilitators’ adaptability in response to low attendance. At Welcome Haven, facilitators noted that online workshops that were originally designed for children and adolescents became a space for mothers to “have a little moment” for themselves when children were not attending.

Most service providers discussed retaining certain online services due to their accessibility even as they expressed enthusiasm for a return to in-person activities. One service provider summarized that online programs had “high impact” but “low output,” considering the substantially less effort and energy in organizing zoom meetings. Nonetheless, when facilitators informed the regular participants of the resumption of in-person Welcome Haven workshops, participants expressed “genuine excitement” and gratitude to return to in-person activities, particularly because they wished to see the facilitators with whom they had built bonds.

In summary, we found that online service delivery, while offering some advantages, was less preferred by community workers and participants alike. Importantly we also learned that in addition to the multiple accessibility barriers to online services, virtual programming also limited much needed interpersonal connection, and left many participants feeling less secure and potentially surveilled. Finally online services posed risks in terms of giving a false sense of emotional safety and limited privacy.

### 3.2. Organizational adaptation to the COVID-19 pandemic

#### 3.2.1. Service adaptation and expansion

Despite the numerous challenges in connecting with asylum seekers during the pandemic, most service providers we interviewed also emphasized the different ways their organizations adapted, even expanded, their services to meet asylum seekers’ needs. One organization known for hosting orientation sessions for only newly arrived asylum seekers saw that during the pandemic, previous participants who were in need of resources and connections returned. This prompted the service provider to “reimagine” the existing program to encourage sustained connections between the organization and these past participants. Propelled by increasing demand for wellness services as a result of the pandemic, another organization dedicated resources to expand their psychosocial service provision:

As a community organization, you have to use your funds adequately. You have to balance resources and there are different needs [in] different sectors. So, where [mental health support] fits in was always kind of difficult to push, but then […] COVID hit and we were like, okay, this can't really be postponed anymore. There's an immediate need for it, so we worked hard to structure it, push it forward.

By sharing how difficult it was to integrate mental health support into the organization mandate pre-pandemic due to limited funds, this community organizer described how they were able to seize the opportunity provided by COVID-19. Similarly, another organization that traditionally provided in-person services took advantage of a decreased demand for services as a result of border closures to prioritize developing an online multilingual welcome guide for newcomer families. A service provider shared that the guide was widely disseminated within the newcomer community and helped establish more partnerships and garner publicity. This organization also developed new approaches for reaching hard-to-reach refugee claimants such as a telephone helpline, and several online chatting forums (WhatsApp or Viper).

Nonetheless, organizations faced profound challenges in developing new spheres of expertise and securing the human and financial resources needed to sustain their services. While many organizations noted receiving emergency funds because of the pandemic, some organizations initially lost funding they had previously secured because the funds were redirected to other emergency initiatives related to the pandemic. Similarly, in the public sector, health and social service providers usually dedicated to provision of refugee claimant services were sometimes, especially in 2020, redeployed to service in eldercare or COVID wards. One service provider shared that the increasing need for online services also demanded more human resources such as more law student interns and non-specialized mental health providers. The organization that started a telephone helpline also explained that the hotline was mainly managed by one staff member who found the work to be highly demanding and difficult to sustain. This service provider noted that her workload was only lightened once public health restrictions eased and more staff were hired.

For several organizations, pivoting and expanding their services to other vulnerablized population was necessary to adapt to the new pandemic context where there were fewer asylum seekers coming in due to border closure but growing issues of food and financial insecurity, family violence, deprivation and social isolation. One organization whose mandate was to provide accommodation to asylum seekers pre-pandemic opened their shelter to include the homeless, indigenous people and those with a precarious status such as undocumented women fleeing from domestic violence. This organization also mentioned receiving so many food donations during the pandemic that they turned their office into a food bank. Similarly, another organization who—.

prior to the pandemic—only supplied free furniture to asylum claimants and those with precarious status, expanded their services to victims of domestic violence who held residency in Canada. However, organizations’ ability to remain flexible was ofttimes contingent on their program mission and funding. Both organizations noted that this ability to pivot would not be possible if they were operating within the public social services sector where funding envelopes are attached to stricter rules about mandates and populations.

Service providers attributed their prompt organizational flexibility to a stance of client-centeredness and being on the frontline to witness claimants evolving predicaments. One service provider explained:

I think a big part [of what we learnt during the pandemic] is […] services [should be] client facing and [by] being with the community […] you hear the experiences they're going through. So, I think it's being continuously connected to the community that is so valuable, […] and to not stick with one type of service or one type of response, but to adapt and change to what is actually needed.

As this participant explained, compared to larger public sector organizations with more rigid bureaucratic constraints, community organizations had significantly more agility and, they felt, direct access to experiences of refugee claimants.

#### 3.2.2. Partnerships and collaborations

An important factor that appeared to support organizations’ ability to adapt to the challenges of the COVID-19 pandemic was the strengthening or establishment of inter-organization collaboration, allowing the leveraging each other’s resources and skills to provide expanded services. For instance, two of the organizations we spoke to worked together to deliver online cooking workshops for asylum seekers, where one offered material resources and the other sent staff to promote, coordinate, and facilitate. Another organization detailed their collaborations with a wide-ranging number of community organizations to facilitate different programs such as yoga, psychotherapy, and art therapy. The close partnerships this organization forged also opened avenues for knowledge exchange that were particularly crucial in advocating for matters relating to health inequities, such as healthcare access, amplified by the pandemic. This organization mentioned hosting “community talks” where they shared information on “different rights, different insurance types” with community partners including private healthcare practitioners and other community organizations to raise awareness and alleviate barriers to service access. Additionally, the solidarity borne during the pandemic was also motivating and valuable to service providers on an interpersonal level:

[One] thing I really appreciate is that one community organizer which I don't work [with], she just called once a week all the organizations in the neighborhood, and she [asked] us, how are you doing? What do you need? How can I help? And you personally? How [are you doing]? And it was just two minutes every week, but I was really grateful, […] I think we had a lot of solidarity between all of us, and we got through this.

This service provider emphasized that close collaboration with other community organizations on both the personal and organizational level was essential in sustaining their work and proved to have long-term impact. These partnerships that encouraged knowledge exchange, advocacy coalitions, and interpersonal relationships continued to strengthen through ongoing collaboration even as COVID-19 restrictions have subsided.

Despite the flourishing of collaborations forged in the strain of responding to COVID-19, building partnerships was not without challenges. For example, during the initial implementation of Welcome Haven, a few community organizations declined to collaborate with us, citing public health regulations, limited human resources (often due to COVID-19 staffing shortages) or funding mandates that excluded asylum seekers and were designated exclusively for immigrants or accepted refugees. This kind of rejection was also reported by another organization during our interviews, making it difficult at times for organizations to refer asylum seekers to other community organizations for specific support. Another challenge to working together was organizations seeking financial compensation for collaborations (such as rent for use of space or honoraria for staff), which limited partnerships when budgets were already stretched. Even with the organizations who were willing to collaborate initially, rising infection rates sometimes elicited anxiety in the leadership of collaborating organizations such that they requested last-minute cancellations of shared workshops or activities even if allowable by public health regulations. Sometimes the Welcome Haven’s views of what to prioritize was in tension with the concerns of a partnering organization. For example, while our team felt requiring proof of vaccination, as requested by one of our partners (when not required by public health for essential services), would limit access to much-needed workshops in already vulnerablized and isolated refugee claimant families, our partners felt it would be protective of families who would be disproportionately disadvantaged if they contracted COVID-19. These negotiations across organizations were at moments tenuous and delicate. It required open, bi-directional conversation to resolve tensions between priorities, all of which did in fact have the wellbeing (and protection) of refugee claimant families at heart. Other conflicts, like the limited mandates of organizations that excluded asylum seekers, were not resolved.

In summary, we found that despite significant challenges to operations during the height of the COVID-19 pandemic in Montreal, organizations swiftly found means to pivot and adapt their services to meet the rapidly shifting needs of refugee claimants. Our findings also suggest that necessity was indeed the progenitor of innovation, and that new partnerships and collaborations were more readily formed during the pandemic, even as these same collaborations were at times delicate and not entirely without tension.

## 4. Discussion

As one of the epicenters of COVID-19 in Canada, Montreal suffered a high incidence and prevalence rate of infection (55), as well as strict public health regulations, which further limited service access for vulnerablized marginalized communities including refugee claimants (56). The current study explores how both service providers and asylum claimants experienced community-based service provision during the Covid-19 pandemic. Their perspectives offer a window into the transition from in-person to online services, and also reveal the challenges and opportunities organizations faced in responding to evolving population needs and vacillating levels of financial and other resources.

While we identified strengths in online activities, our results suggest the need for more critical evaluation of online service delivery by highlighting its significant limitations, especially for the asylum-seeking population. We discovered that informational workshops and individual support were the most appropriate services to offer online for this population. Other types of psychosocial services (especially ones that emphasize social connection) depended on (1) previous in-person rapport; (2) parental supervision (when working with children); and (3) creative means of bridging the social isolation gap while remaining sensitive to the unique needs of this population. Similar to our findings, Hynie and colleagues also found that the transition to virtual mental health support was met with concerns about security and privacy among refugees as well as technological barriers such as connectivity issues and limited digital literacy (57). Hynie highlighted the importance of flexibility to respect the heterogeneous psychosocial needs of newcomers and offer in-person services when requested to improve access to care (57). This recommendation supports our finding that cautions the all-encompassing transition to online programs specifically for the asylum-seeking population and stands in contrast to literature that found comparative level of effectiveness in online versus in-person programs (58). While our results acknowledge the accessibility and convenience online services offer (59), we also found there were real dangers to physical and emotional safety in online spaces, especially in situations of possible family violence. In this sense, poor attendance as well as participants’ reluctance to fully participate, even when present online, could be seen as an appropriate and adaptive coping strategy to mitigate some of the safety risks of online forums, including ones designed specifically for their needs. As our results suggest, online activities with children might inadvertently perpetuate threats to emotional safety as a result of facilitators and participants’ confusion over online functions which at times hindered communication and resulted in silencing. Another Canadian study echoed our recommendation by similarly concluding that attention should be given to keeping in-person community services open for newcomers during public health emergencies (32).

In terms of organizational functioning, we learned, in keeping with other research (60), that flexibility and an ability to quickly adapt were key features associated with program success. Compared to some public institutional services which are bound by bureaucratic processes that delay decision-making and changes in procedure, practices and service offerings, our study suggests the resourcefulness of community organizations is largely due to their bottom-up approaches and organizational latitude to re-allocate financial and other resources. Similar collaborative and ‘bottom up” approach was taken by faith-based and religious communities in Montreal during COVID, which facilitated recognition of community needs and promoted more inclusive and effective public health policies (61).

This said, the work of community-based organizations was not without strain. As we learned, limited financial and human resources increased burden of service providers and partnerships between community organizations required continuous conversations, at times eliciting tensions and disagreements on how to approach COVID restrictions and best protect asylum seekers from infection risk while mitigating the lack of access to social and instrumental support. These negotiations are, nonetheless, crucial to the implementation and sustainable delivery of essential services to the asylum-seeking community and thus deserve further research attention.

Our findings bring to mind the ecosocial ADAPT framework which encapsulated psychosocial pillars that require restoration for individuals and communities affected by war and violence. In his ADAPT model, Silove describes five domains as critical determinants of asylum seekers’ wellbeing and recovery during resettlement: (i) safety and security, (ii) bonds and networks, (iii) justice, (iv) role and identity, and (v) existential meaning (49). Our findings highlighted how community organizations sought to restore these pillars in their online service delivery and organizational adaptation during the pandemic. Past research had found social connectedness to be a key determinant of asylum seekers’ mental health (62). For example, enduring longer periods awaiting family reunification, not having access to family support or local connections and facing discrimination due to a rise in xenophobia contributes to feelings of loneliness and is associated with higher levels of depression among this population (17, 18, 63). Therefore, psychosocial workshops with the aim of bridging social connection may protect claimants’ mental health through restoration of bonds and networks. As some interviewees noted, such connections were also essential to the recovery of existential meaning, or, as they described, feeling like they “existed.” Further, in facilitating service access and knowledge exchange, the work of community organizations also seems to protect safety and justice for claimants by promoting inclusion and equity. For refugee claimants who already face formidable barriers to accessing healthcare services before the pandemic, the *embodiment* of exclusion that the pandemic (re)produced may continue to drive decrements in their health status unless redressed (64). As we have seen, community organizations approach the restoration of justice as a core part of their work. As such, community organizations play an essential role in protecting the health and wellbeing of asylum seekers and fought hard to resist their services from being relegated to the margins during the pandemic.

One limitation of the study is its small sample size of 9 service providers and 13 refugee claimants. The constantly changing Covid restrictions and protocols made it challenging for community workers to devote time to participating in research. Nonetheless, for qualitative studies, modest sample sizes are not uncommon and can still provide a rich, contextualized data sets to illuminate how phenomenon are experienced and understood by the people closest to them (65, 66). The refugee claimants who participated were also all women. Thus, our study does not capture the experiences of fathers, or single men who may indeed have experienced the services provided during COVID differently, given known gendered differences in help-seeking. Our study was also set in Montreal, a city with a public healthcare system, and its own specific ecosystem of community organizations. While the situation in Montreal may be distinct, and not generalizable to all contexts, many of the central findings are likely relevant in many HICs and large cities receiving refugee claimants who faced similar COVID public health restrictions and challenges to providing services.

## 5. Conclusion

From understanding the experiences of community-based organizations that serve refugee claimants during the COVID-19 pandemic in Montreal, this study illuminated the limitations of online service delivery and the essential role of these organizations in claimants’ resettlement. Given that asylum seekers confront significant barriers to service access despite experiencing a disproportionate burden of stressors and health risks related to their immigration status, they are especially reliant on community organizations for support. Implementing community-based strategies to provide support have shown to be essential in facilitating access to health and social services and promoting the psychological well-being of migrant communities in various contexts (67, 68). Alongside infection control measures, it is crucial that community organizations maintain in-person services so that healthcare and social services remain equitable and accessible. We hope that the results of this study will guide decision makers, community groups and care providers to rethink the place of community organizations and consequently develop improved policies that protect these organizations and preserve what are clearly *essential* services for refugee claimants in high income countries such as Canada. Although the pandemic amplified pre-existing structural inequalities, particularly among marginalized populations, it also presents an opportunity to learn from our mistakes so as to implement resilient structures and programs that are more resistant to system failure during public health emergencies.

## Data availability statement

Excerpts of de-identified transcripts relevant to the study may be made available upon request, excluding any which might compromise participant confidentiality.

## Ethics statement

The studies involving human participants were reviewed and approved by the Research Ethics Committee of the CIUSSS du Centre- Ouest-de-l’Île-de-Montréal (Project 2021-2461). The participants provided their written or oral informed consent to participate in this study.

## Author contributions

YW and RaK: in-depth data analysis, conceptualization and methodology. YW, RoK, and AA: data collection and initial data analysis. YW, AA, and LK: preparation of original draft. RaK: principle investigator and in-depth revision of manuscript. All authors have participated in the revision and approved the final version of the manuscript submitted for publication.

## Funding

RaK received funding support for this research through the Social Sciences and Humanities Research Council (SSHRC) and the Réseau Québécois COVID-Pandémie; YW received funding support from Canadian Graduate Scholarship - Masters (CGSM) and Fonds de recherche Québec - Société et culture (FRQSC).

## Acknowledgments

We would like to acknowledge the workshop facilitation and fieldnotes contributed by research assistant Gabriela Peterson and drama therapists Mau Caron and Sophie Chappell as well as the invaluable contributions of participants, both refugee claimants and community organizations.

## Conflict of interest

The authors declare that the research was conducted in the absence of any commercial or financial relationships that could be construed as a potential conflict of interest.

## Publisher’s note

All claims expressed in this article are solely those of the authors and do not necessarily represent those of their affiliated organizations, or those of the publisher, the editors and the reviewers. Any product that may be evaluated in this article, or claim that may be made by its manufacturer, is not guaranteed or endorsed by the publisher.
